# Transcriptome Characterization and Identification of Chemosensory Genes in the Egg Parasitoid *Anastatus orientalis*, Along with Molecular Cloning, Sequence Analysis, and Prokaryotic Expression of the Odorant Binding Protein 8 (*AoOBP8*) from *A. orientalis*

**DOI:** 10.3390/insects16111117

**Published:** 2025-10-31

**Authors:** Xinyu Liu, Yanyan Bai, Yu Qi, Baozhi Liu, Yingying Zhao, Yuting Wu, Jiating Yang, Yanan Wang, Shouan Xie

**Affiliations:** Key Laboratory of National Forestry and Grassland Administration on Management of Forest Bio-Disaster, College of Forestry, Northwest A&F University, Yangling 712100, China; xinyuliu2023@nwafu.edu.cn (X.L.); baiyanyan@nwafu.edu.cn (Y.B.); qiyu427@nwafu.edu.cn (Y.Q.); liubaozhi2024@163.com (B.L.); zhaoyingying@nwafu.edu.cn (Y.Z.); wyt261888@163.com (Y.W.); 15333443128@163.com (J.Y.); wynnnqz@gmail.com (Y.W.)

**Keywords:** *Anastatus orientalis*, *Lycorma delicatula*, transcriptome analysis, chemosensory-related gene, biological control

## Abstract

**Simple Summary:**

*Anastatus orientalis* is an important natural enemy of *Lycorma delicatula* and is being tested as a potential biological control agent for invasive *L. delicatula* around the world. Studying the genes related to chemosensory proteins in parasitic wasps is vital for enhancing their ability to locate hosts and improve parasitism efficiency. In this study, transcriptome sequencing was used to identify 62 chemosensory-related genes in male and female adults from *A. orientalis*. The values for fragments per kilobase per million (FPKM) indicated that the chemosensory protein gene families in *A. orientalis* exhibit different expression levels in male and female adults, with some genes showing significant differences and displaying sex-biased expression. Meanwhile, the nucleotide and amino acid sequences of *AoOBP8* and the physiochemical properties of its encoded protein were clarified. The gene *AoOBP8* was expressed in male and female adults and various tissues of adults, and the highest expression level was detected in the head. The study showed that *AoOBP8* played a significant role in olfactory recognition in *A. orientalis*. These results provide molecular evidence supporting further exploration of chemosensory mechanisms governing host recognition and localization in *A. orientalis*.

**Abstract:**

*Anastatus orientalis*, a prominent egg parasitoid of *Lycorma delicatula*, demonstrates considerable potential for biological control. *A. orientalis* is dependent on host volatiles to identify and locate appropriate hosts for reproduction, with its olfactory system playing a vital role in volatile detection. There is little known about the chemosensory genes in *A. orientalis*. Therefore, here, we conducted a transcriptome analysis of the males and females from *A. orientalis*. Overall, 24 odorant binding proteins (OBPs), 4 chemosensory proteins (CSPs), 26 odorant receptors (ORs), 3 gustatory receptors (GRs), 3 ionotropic receptors (IRs), and 2 sensory neuron membrane proteins (SNMPs) were identified by transcriptome analysis. The values for fragments per kilobase per million (FPKM) indicated that the chemosensory protein gene families in *A. orientalis* exhibit different expression levels in male and female adults, with some genes showing significant differences and displaying sex-biased expression. Furthermore, RACE technology, phylogenetic analysis, and expression analysis were used to investigate the role that *AoOBP8* plays in olfaction in *A. orientalis*. *AoOBP8* was highly expressed in females and the heads of adults, indicating that the gene has a crucial role to play in the search for hosts and in oviposition in female adults, while the head is crucial in recognizing chemical information. These results contribute to a deeper understanding of the functions of chemosensory protein gene families in *A. orientalis* and offer a reference for developing biocontrol methods for forestry pests.

## 1. Introduction

The spotted lanternfly (hereafter SLF), *Lycorma delicatula* (White) (Homoptera: Fulgoridae), is a prevalent garden pest with a broad distribution [1]. It poses a significant threat to economically important tree species and ornamental plants, affecting both domestic and international environments [2]. In regions where the SLF is already established, various control measures have been implemented, including the use of insecticides [3], traps [4], and chipping to destroy egg masses [5]. However, these efforts appear to be insufficient given that the SLF is still spreading rapidly to new regions [6].

Parasitic wasps, as an important type of natural enemy insect in biological control, play a significant role in the green control of agricultural and forestry pests [7]. *Anastatus orientalis*, as the native parasitic wasp of *L. delicatula*, possesses considerable potential for biological control [8,9]. *A. orientalis* is a dominant egg parasitoid wasp of *L. delicatula*. It was first discovered in China in 2013 and was formally described and published in 2015 [8]. In China, the distribution pattern of *A. orientalis* is the same as that of *L. delicatula* [10]. It is a key biological factor in controlling the population of the *L. delicatula*, capable of accurately locating and identifying its egg masses for parasitism [11]. Under natural conditions, the parasitism rate of *A. orientalis* can reach up to 40.2%, and up to 77.3% in laboratory artificial breeding conditions [7]. Moreover, *A. orientalis* is easy to rear in captivity and has strong host-seeking abilities. Bao et al. showed that the parasitism rate of *A. orientalis* decreased with the increase in host densities of *Antherea pernyi* Guérin and Méneville, and the daily maximum parasitism amount on *A. pernyi* was 16.2%, which demonstrated the high biocontrol potential of *A. orientalis* [9]. In other words, *A. orientalis* has demonstrated considerable potential for reducing *L. delicatula* populations, and the research opportunities surrounding this natural enemy are exceptionally promising. Consequently, conducting research on *A. orientalis* is essential.

Chemoreception is critical for the survival of insects. Insects display a variety of behavioral responses, such as mating, host searching and ovipositing, in response to different odor signals detected in their living environment [12,13,14]. Odorant recognition in insects involves multiple olfactory-related proteins, including odorant binding proteins (OBPs) and chemosensory proteins (CSPs), ionotropic receptors (IRs), odorant receptors (ORs), gustatory receptors (GRs), and sensory neuron membrane proteins (SNMPs) [14,15]. Understanding the molecular mechanisms of insect chemosensory proteins provides valuable insights into their olfactory system, offering potential avenues for regulating insect behavior. Previous studies suggested that the success of parasitic wasps in suppressing pest populations depends on their ability to locate hosts in a complex chemical environment [7,16]. Like most insects, parasitic wasps locate their hosts by foraging, and reproduction occurs through a series of behavioral activities, regulated mainly by chemoreception [17,18,19]. The identification of chemosensory genes in parasitic wasps is crucial, both to address the mechanisms controlling intraspecific or interspecific chemical communication and for the potential genetic manipulation of parasitoid behavioral responses via the modification of their ability to discriminate the chemical cues used in biological control strategies [20]. Currently, there are no reports on the molecular mechanisms of chemosensory proteins in *A. orientalis*. Therefore, conducting research on the identification and expression patterns of genes related to chemosensory proteins in *A. orientalis* is crucial.

In recent years, with the advancement of technology, the identification of genes associated with chemosensory proteins in many parasitic wasps has been successfully accomplished using transcriptome sequencing and high-throughput sequencing technologies, such as *Chouioia cunea* [14], *Aulacocentrum confusum* [15], *Cotesia vestalis* [21], *Anastatus japonicus* Ashmead [22], *Sclerodermus* sp. [23], and *Telenomus remus* Nixon [24], providing a foundation for understanding chemosensory protein mechanisms in insects. However, little is known about the transcriptomic studies or the olfactory molecular mechanisms of *A. orientalis*. Therefore, studying the molecular characterization of the odorant sensory mechanisms in *A. orientalis* is of particular importance.

The protection and utilization of native parasitic insects is an important measure for pest control, and *A. orientalis*, as a newly discovered local parasitic insect group in China, plays a significant role in controlling *L. delicatula*. At present, research on *A. orientalis* is mainly focused on its morphology, parasitic efficiency, group rearing techniques and control effectiveness [8,9,25]. However, studying the molecular characterization of the odorant sensory mechanisms in *A. orientalis* is highly significant for improving its parasitism rate in the field. But there is no report on the molecular mechanism of chemical recognition in *A. orientalis*. Therefore, high-throughput sequencing was employed to analyze the transcriptomes of male and female *A. orientalis* adults, and to identify and analyze chemosensory protein genes in this study. These results contribute to our understanding of the olfactory mechanisms in *A. orientalis* and offer a theoretical foundation for formulating sustainable and eco-friendly pest management strategies.

## 2. Materials and Methods

### 2.1. Insect Collection

The *A. orientalis* specimens used in this study were collected from parasitized egg masses of *L. delicatula* on 9 December 2023 in Xi Lijiagou Village (34°15′ N, 109°45′ E), Puhua Town, Lantian County, Xi’an City, Shaanxi Province, in China. After the emergence of parasitic wasps, adults were fed with a 10% honey water solution. Male and female adults were separated according to morphological characteristics, and the heads with antennae, thorax, and abdomen of the *A. orientalis* adults were dissected for RNA extraction. For each treatment, there were 3 biological replicates. All collected tissues were placed in 1.5 mL Eppendorf tubes, frozen immediately with liquid nitrogen, and then stored at −80 °C until use.

### 2.2. RNA Extraction and cDNA Library Construction and Transcriptome Sequencing

To fulfill the sample size requirements for transcriptome sequencing, ensuring that each sample weighed no less than 100 mg, we established three biological replicates for both the female and male adult groups of *A. orientalis*, resulting in a total of six samples, namely, AoF1, AoF2, AoF3, AoM1, AoM2, and AoM3. Each of these samples contained 100–120 individual parasitic wasps. RNA was extracted from the above-mentioned six samples by using the Eastep^®^ Super total RNA Extraction Kit (Cat.# LS1040, Shanghai Promega Trading Co., Ltd., Shanghai, China). Once the concentration and purity of the total RNA extracted from *A. orientalis* adults were verified, construction and sequencing of the cDNA library were performed (Supplementary Document S1) by Wuhan Maiwei Metabolic Biotechnology Co., Ltd. (Wuhan, China).

### 2.3. Transcriptome Assembly, Gene Annotation, Identification and Sequence Analysis

Once clean and high-quality data was obtained, DIAMOND software (v2.0.4) was used to align the unigene sequences with NR, Swiss-Prot, COG, KOG, eggNOG4.5, and KEGG databases [13,26,27], KEGG orthology results for unigene were obtained using KOBAS, and InterProScan was used to analyze the GO orthology of novel genes via the integrated InterPro database [28,29,30]. Following the prediction of unigene amino acid sequences, HMMER software (v3.1b2) was utilized to align them with the Pfam database for annotation purposes [31,32].

Regarding the analysis of transcriptome data of *A. orientalis* adults, all candidate OBPs, CSPs, ORs, GRs, IRs, and SNMPs were manually checked using the BLASTx program (https://blast.ncbi.nlm.nih.gov/Blast.cgi, accessed on 6 July 2025), with alignment parameters including species name, gene description, accession number, E-value, and identity, leading to the identification of chemosensory proteins. To compare the differential expression of chemosensory genes in the transcriptomes of male and female *A. orientalis*, the read number for each chemosensory gene between male and female adults was converted to RPKM (Reads Per Kilobase per Million mapped reads) [33,34].

Based on the results of transcriptome data screening and the results of the preliminary experiments, we selected the *AoOBP8* gene for further analysis, as it demonstrated high expression levels in our preliminary experiments.

### 2.4. Molecular Cloning and Prokaryotic Expression of the Odorant Binding Protein 8 of A. orientalis (AoOBP8)

#### 2.4.1. RNA Isolation and AoOBP8 cDNA Synthesis

First, total RNA was extracted from each sample using the Eastep^®^ Super total RNA Extraction Kit (Cat.# LS1040, Shanghai Promega Trading Co., Ltd., Shanghai, China). Once the concentration and purity of the total RNA extracted from *A. orientalis* had been verified, total RNA was reverse-transcribed using the PrimeScript^TM^ II 1st Strand cDNA Synthesis Kit (Code No. 6110A, Takara Biomedical Technology (Beijing) Co., Ltd., Beijing, China). The detailed method can be found in Supplementary Document S1.

#### 2.4.2. Molecular Cloning

Fragments of the putative *AoOBP8* gene were procured from the transcriptome database for *A. orientalis*. The veracity of the sequences was established by polymerase chain reaction (PCR) using the primers in Table 1. Full-length cDNA was obtained by 5′- and 3′-RACE using the SMARTer^®^ RACE5′/3′ kit (Code No. 634858/59, Takara Biomedical Technology (Beijing) Co., Ltd., Beijing, China) with the specific primers listed in Table 1. We subsequently recovered and purified the PCR product. The purified DNA was ligated onto the PGEM-Teasy Vector (Cat.# A1360, Shanghai Promega Trading Co., Ltd., Shanghai, China) and the dideoxynucleotide method was used for sequencing (Sangon Biotech, Shanghai, China).

#### 2.4.3. Bioinformatic and Phylogenetic Analyses

The cDNA sequence of *AoOBP8* was translated with the Translate tool (https://web.expasy.org/translate/ (accessed on 6 July 2025)). Amino acid sequences were deduced using ExPASy (http://web.expasy.org/translate (accessed on 6 July 2025)). The molecular weight (MW) and isoelectric point (pI) of the deduced amino acid sequences were predicted with Compute pI/Mw (https://web.expasy.org/protparam/ (accessed on 6 July 2025)). The N-linked glycosylation sites were analyzed using NetNGlyc 1.0 Server (http://www.cbs.dtu.dk/services/NetNGlyc/ (accessed on 6 July 2025)). Sequence comparisons were performed using DNAMAN. Additionally, a phylogenetic tree was constructed using a total of 20 insect TPS protein sequences obtained from NCBI via MEGA7.0 software and Clustal X 1.83 using the maximum likelihood method. Bootstrap values were calculated based on 1000 replicates [35].

#### 2.4.4. Expression of AoOBP8 Gene

cDNA templates derived from different developmental stages of *A. orientalis* were used for temporal expression tests. Primers were designed for quantitative real-time PCR (RT-qPCR) by Prime 5.0 and are listed in Table 1. The expression of the target gene was measured by RT-qPCR and normalized with two stable reference genes (β-Actin and α-tubulin). Detailed reaction systems for each PCR can be found in Supplementary Document S1. The reactions were performed with a StepOnePlus^TM^ Real-Time PCR Instrument (Thermo Fisher Scientific, Singapore). The relative expression level was calculated using the 2^−ΔΔCt^ method [36].

#### 2.4.5. Prokaryotic Expression and Identification of His-AoOBP8

The expression primers corresponding to the gene encoding *AoOBP8* were designed using Prime 5.0 software, and EcoRI and HindIII enzyme cutting sites were introduced at the 5′ and 3′ ends, respectively. The expression fragment of β-enolase was amplified using EX Taq DNA polymerase, and the PCR product was verified by sequencing. The expression fragment and the pET-30a plasmid were double-digested separately and then ligated by T4 DAN ligase, and then the expression plasmid, pET-30a-β-enolase, was constructed successfully. Afterward, the expression plasmid was introduced into *E. coli* DH5α cells for expansion, followed by extraction using the plasmid mass extraction kit (Tiangen, Beijing, China).

The expression plasmid was transformed into *Escherichia coli* Origami (DE3) cells via heat shock, and positive clones were selected for protein expression. *E. coli* Origami (DE3) cells carrying the expression plasmid were incubated at 37 °C for 4 h with shaking until cultures reached an optical density (OD) = 0.6. The synthesis of β-enolase was induced with the addition of isopropyl β-d-thiogalactoside (IPTG; final concentration of 1 mM) to the cultures, and the cells were collected by centrifugation after incubation at 20 and 37 °C for 4 h, respectively. The cells were disrupted by ultrasonication and subsequently centrifuged to separate the soluble and insoluble fractions. The soluble and insoluble fractions were analyzed by reduced sodium dodecyl sulfate polyacrylamide gel electrophoresis (SDS–PAGE).

About Western blot detection of the expressed fusion protein His-AoOBP8. First, place the PVDF membrane in 200 mL of blocking solution consisting of 5% skim milk powder, and seal it overnight at 4 °C. The following day, wash the membrane with 30 mL of TBST buffer and drain it after washing. Next, soak the membrane in a blocking solution (5% skim milk powder) diluted with mouse anti-6 × His primary antibody at a volume ratio of 1:1000, and incubate it overnight at 4 °C. The next day, wash the membrane six times with 30 mL of TBST buffer, allowing 5 min for each wash. After completing the sixth wash, drain the membrane and soak it in goat anti-mouse IgG secondary antibody diluted with blocking solution at a volume ratio of 1:3000. Incubate at room temperature for 2 h, followed by another six washes with 30 mL of TBST buffer for 5 min each. After washing, take photographs and observe the results after color development using the WTMB color reagent kit.

The crude protein of the His-AoOBP8 fusion protein was purified using a Ni-NTA affinity chromatography column. The equilibrium column was washed with imidazole elution solutions at concentrations of 20, 50, and 500 mmol/L. Subsequently, the protein was eluted through overnight dialysis with Tris HCl buffer at pH 7.4 and 50 mmol/L. Finally, analyze the collected protein samples using 15% SDS-PAGE and validate the purified fusion protein through Western blot.

### 2.5. Statistical Analysis

The data were summarized as the mean ± SE (standard error) for all data sets. A one-way analysis of variance (ANOVA) was performed on data with more than 3 groups using SPSS 26.0. Differences between means were tested using a Student-Newman-Keuls (S-N-K) test for multiple comparisons. The independent sample *t*-tests were used for data with fewer than 3 groups. All experiments were performed with 3 biological replicates. Each biological replicate was performed with 3 technical repetitions. Differences were considered statistically significant at the 5% level (*p* < 0.05).

## 3. Results

### 3.1. Transcriptome Sequencing and Assembly of A. orientalis

We obtained a total of 46.44 Gb of high-quality reads, with each sample yielding more than 6.41 Gb of clean data. The Q30 base percentage exceeded 97.03%, and GC content ranged from 36.03% to 38.01% (Table 2). Transcript assembly using Trinity produced 71,513 transcripts with an N50 length of 2692 bp. The analysis of the assembled sequences yielded 40,182 unigenes, with an N50 length of 2904 bp, reflecting strong assembly integrity (Table 3).

### 3.2. Functional Annotation of the Unigenes in A. orientalis

The unigene sequences were aligned using Diamond 4.6.8 software against eight major databases: KEGG, NR, Swiss-Prot, TrEMBL, KOG, GO, and Pfam. The transcriptome data of *A. orientalis* adults produced a total of 40,182 annotated unigenes, with the NR and TrEMBL databases providing the highest number (26,113 unigenes, 64.99%, 26,326 unigenes, 65.52%, respectively) (Table 4). Meanwhile, the results indicated that 28,075 of the 40,182 unigenes (69.87%) had been annotated in at least one database.

**Figure 1:**
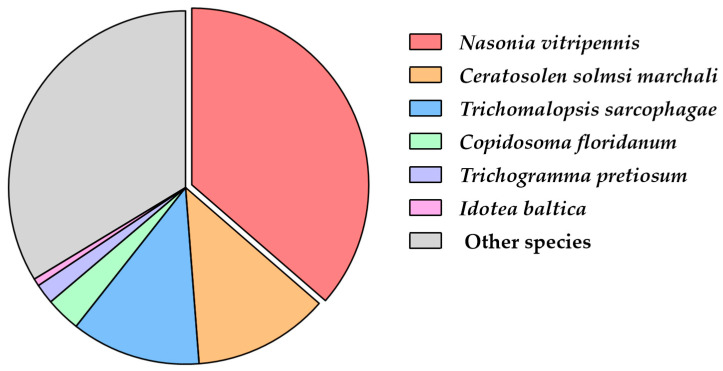
Species distribution of unigenes of *A. orientalis* in NR database.

Based on the annotation results from the NR database, a species distribution map of the sequence alignment was drawn. The results showed that 36.38% of the genes were aligned to *Nasonia vitripennis*, 12.4% were aligned to *Ceratosolen solmsi* marchali, 11.84% were aligned to *Trichomalopsis sarcophagae*, 3.18% were aligned to *Copidosoma floridanum*, 1.84% were aligned to *Trichogramma pretiosum*, 0.69% were aligned to *Idotea baltica*, and 33.67% were aligned to other species (Figure 1).

### 3.3. Functional Classification of Unigenes in A. orientalis

#### 3.3.1. GO Enrichment Analysis

**Figure 2:**
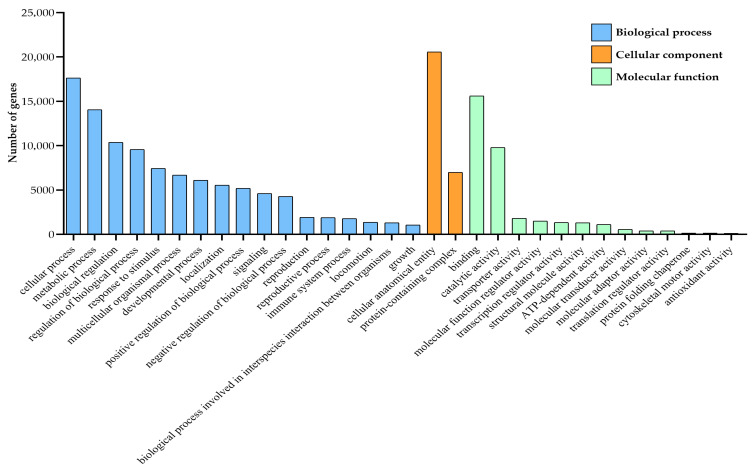
Gene ontology classifications of the *A. orientalis* unigenes.

In the GO database, the gene functions of *A. orientalis* were classified into three categories: biological process (BP), cellular component (CC), and molecular function (MF). Among them, the number of unigenes involved in biological processes was the largest. Cellular and metabolic processes were the two largest groups in both data sets among the biological process category. In molecular function, the number of unigenes involved in binding function and catalytic activity was the highest, with 15,597 and 9786, respectively. In cellular components, the number of unigenes for cell anatomical entities was the largest, at 20,556, followed by protein complexes (6993). In the biological process, the number of unigenes involved in intracellular processes (17,636) and metabolic processes (14,048) was relatively large (Figure 2). These GO annotations provide insights into the global gene expression profile for male and female adults of *A. orientalis*.

#### 3.3.2. KEGG Enrichment Analysis

**Figure 3:**
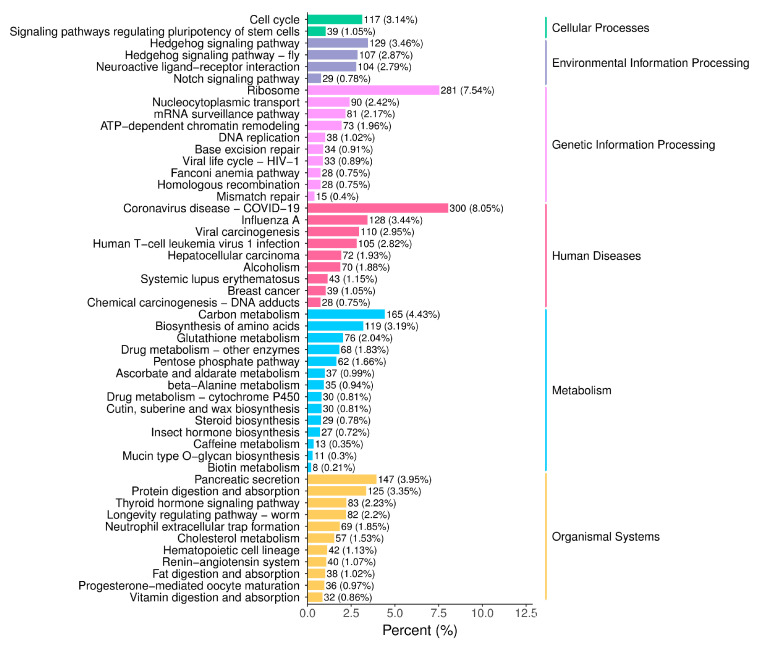
KEGG pathway enrichment column diagram of *A. orientalis*.

**Figure 4:**
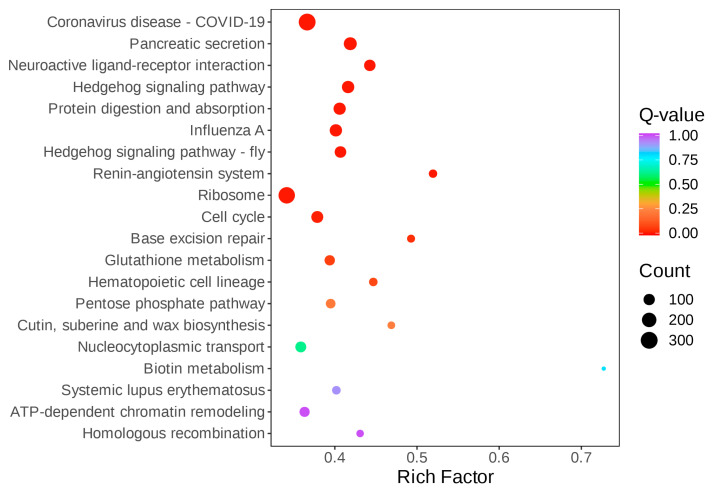
KEGG pathway enrichment scatterplot of *A. orientalis*.

The gender-differentiated expression genes of *A. orientalis* were enriched in six sections, including cellular processes, environmental information processing, genetic information processing, human diseases, metabolism, and organismal systems. A bar chart of enrichment items was drawn, as shown in Figure 3. These pathways include cell cycle (117, 3.14%), ribosome (281, 7.54%), coronavirus disease (300, 8.05%), and carbon metabolism (165, 4.43%), etc. At the same time, the 20 most significantly enriched pathway items were plotted as a scatter plot, as shown in Figure 4. The larger the circle, the higher the abundance. The closer the color is to red, the greater the significance. The trends presented in Figure 3 and Figure 4 are consistent.

### 3.4. Analysis of Differentially Expressed Genes and Identification and Analysis of Candidate Chemosensory Protein Genes

**Figure 5:**
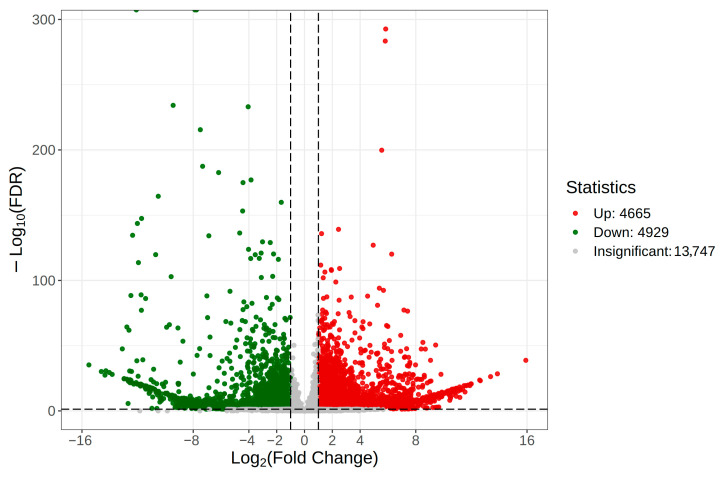
Volcano map of differentially expressed genes of *A. orientalis*.

By analyzing the differentially expressed genes (DEGs) in female and male adults of *A. orientalis*, a total of 13,747 differentially expressed genes were obtained, among which 4929 were down-regulated genes and 4665 were up-regulated genes (Figure 5). Overall, 62 genes related to chemical perception in *A. orientalis* were screened from the database, including 24 OBPs, 4 CSPs, 26 ORs, 3 IRs, 3 GRs, and 2 SNMPs. The identified odor-related genes exhibited varying FPKM values between male and female adult insects. For instance, some genes, such as *AoOBP1*, *AoOBP4*, *AoOBP7*, *AoOBP11*, *AoOBP24*, *AoCSP4*, *AoOR12*, *AoOR13*, and *AoSNMP1*, were expressed at higher levels in male adults compared with female adults. There were also two genes that were exclusively expressed in male adults: *AoOR12* and *AoOR13*. Conversely, other olfactory genes, such as *AoOBP5*, *AoOBP14*, *AoOBP17*, *AoOBP18*, and *AoSNMP2*, demonstrated greater expression levels in female adults than in their male counterparts (Table S1).

### 3.5. Molecular Cloning and Prokaryotic Expression of AoOBP8

#### 3.5.1. Cloning and Sequence Analysis of AoOBP8

**Figure 6:**
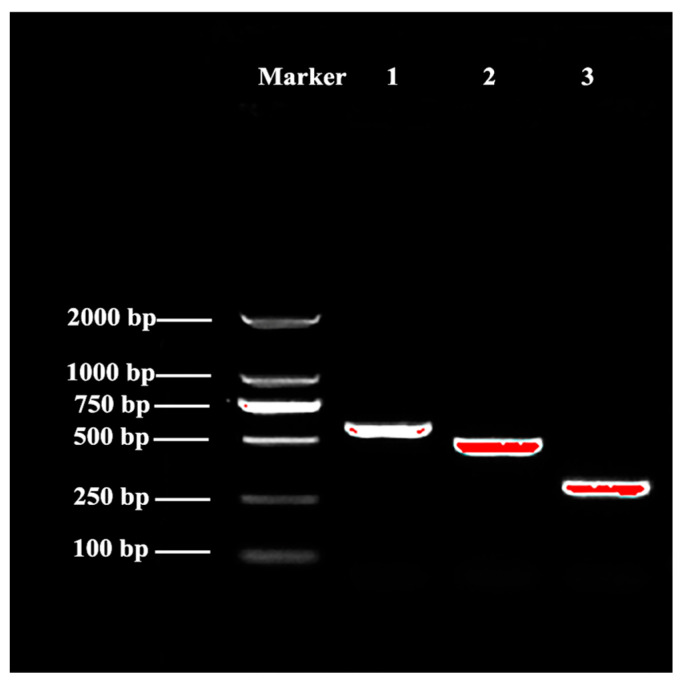
PCR products of *AoOBP8*. Marker represents DL 2000 DNA Marker; 1 represents the middle segment of *AoOBP8*; 2 represents the 5′ RACE segment of *AoOBP8*; 3 represents the 3′ RACE segment of *AoOBP8*.

**Figure 7:**
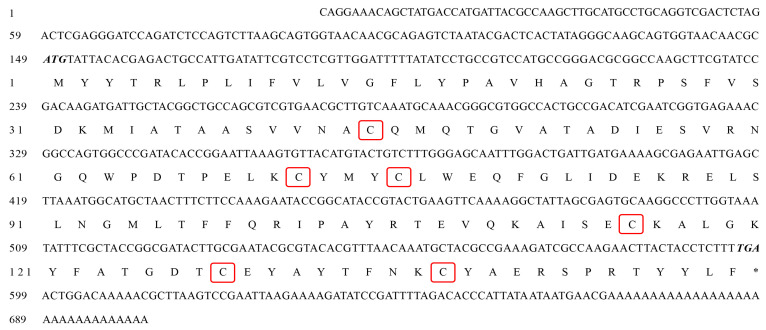
Nucleotide and amino acid sequences of *AoOBP8*. The start and stop codons are indicated in bold italic font, and “*” represents a stop codon. The conserved cysteines are indicated in a red box.

The full-length cDNA sequence of *AoOBP8* was cloned and deposited in the GenBank database (GenBank accession number: PQ836618). The product was analyzed by agarose gel electrophoresis (Figure 6). Our results show that the full length of *AoOBP8* from *A. orientalis* was 701 bp, and the ORF of *AoOBP8* was 450 bp, encoding 149 amino acids (Figure 7). The start codon (ATG) was located at 149 bp and the stop codon (TAG) was located at 599 bp. The sequence included a 5′ untranslated region of 148 bp in length and a 3′ untranslated region of 103 bp in length. The 3′UTR region contained the typical polyadenylation signal sequence AATAA and a 31 bp PolyA tail. The NCBI conserved domain online analysis indicated that the amino acids encoded by the *AoOBP8* had a conserved domain of the insect odorant-binding protein family PBP-GOBP superfamily at positions 91 to 420 (E-value: 3.60 × 10^−20^). Physicochemical properties of the protein coded by *AoOBP8* were as follows: The molecular formula of its protein was derived as C_776_H_1174_N_194_O_219_S_11_. The molecular mass was 17.078 kDa, and the isoelectric point (PI) was 7.53. The total number of negatively charged amino acid residues (Asp + Glu) was 14, and the total number of positively charged amino acid residues (Arg + Lys) was 15. The grand average of hydropathicity was −0.074, indicating that the protein was a hydrophilic protein. The aliphatic index was 74.03 and the instability index was 47.01, suggesting that it was an unstable protein.

In the prediction of hydrophobicity results on the ProtScale online platform of ExPASy, hydrophilic amino acids have negative values, while hydrophobic amino acids have positive values. The higher the absolute value, the stronger the hydrophilicity/hydrophobicity. The results are shown in the following figure. The prediction results of the ProtScale online software (https://web.expasy.org/protscale/, accessed on 6 July 2025) indicate that in the amino acid sequence of *AoOBP8*, hydrophilicity and hydrophobicity are evenly distributed in a mixed manner throughout the entire polypeptide chain. The minimum hydrophobicity value is approximately −2.244, and the maximum value is approximately 3.278. Based on this, it can be preliminarily determined that this protein is a hydropathic (Figure S1).

The predicted signal peptide of the deduced protein suggested a VHA-GT cleavage site between pos. 22 and 23 (probability: 0.9563) (Figure S2). Transmembrane prediction using the hidden Markov models (TMHMM) program result showed that *AoOBP8* has no transmembrane domain (Figure S3). The secondary structure analysis of the SOPMA protein revealed that the secondary structure of the *AoOBP8* protein consists of an alpha helix (65.77%), extended strand (3.36%), and random coil (30.87%). Thus, it can be seen that the alpha helix is the main structural form of the *AoOBP8* protein (Figures S4 and S5).

#### 3.5.2. Sequence Alignment and Phylogenetic Analysis of AoOBP8

Sequence alignment and phylogenetic analysis indicated that the sequence identity of *AoOBP8* from *A. orientalis* with other selected Hymenoptera insect OBPs is 73.03%. The result showed that *AoOBP8* possesses six conserved Cys residues, matching the pattern of classic OBPs (Figure S6). The phylogenetic tree based on OBPs from *A. orientalis* and other species from Hymenoptera was constructed using the neighbor-joining method (Figure S7). The *AoOBP8* in *A. orientalis* was clustered closely with the *NvOBP69a* in *N. vitripennis* with 41% confidence, indicating that the genetic distance between *AoOBP8* and *NvOBP69a* is the shortest. This is consistent with the results of the amino acid sequence homology analysis.

#### 3.5.3. Expression of AoOBP8 in Male and Female Adults and in Various Tissues

**Figure 8:**
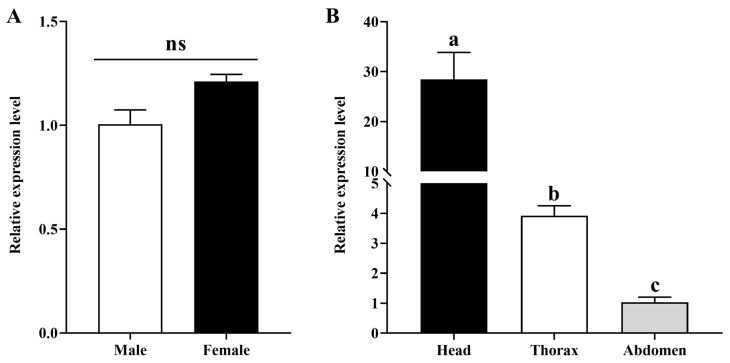
Relative expression level of *AoOBP8*. (**A**) relative expression level in male and female adults, (**B**) relative expression level in different issues (head, thorax and abdomen). The “ns” on the bar chart indicates no significant difference (*p* > 0.05, independent samples *t*-test). While different lowercase letters (a, b, c) on the bar chart indicate significant difference between head, thorax and abdomen (*p* < 0.05, S-N-K test).

The expression pattern of *AoOBP8* was investigated by RT-qPCR (Figure 8). The analysis of the results revealed that the expression level of *AoOBP8* in female adults was slightly higher than that in male adults, but there was no significant difference between them (*p* = 0.055) (Figure 8A). The relative expression level of *AoOBP8* was detected in the head, thorax and abdomen. The relative expression level of *AoOBP8* in various tissues went from high to low in the order of head, thorax and abdomen, with the relative expression level in the head significantly higher than in the thorax and abdomen (*p* = 0.001). The expression levels of *AoOBP8* in the thorax and abdomen were not significantly different (*p* = 0.539) (Figure 8B).

#### 3.5.4. Prokaryotic Expression of AoOBP8

**Figure 9:**
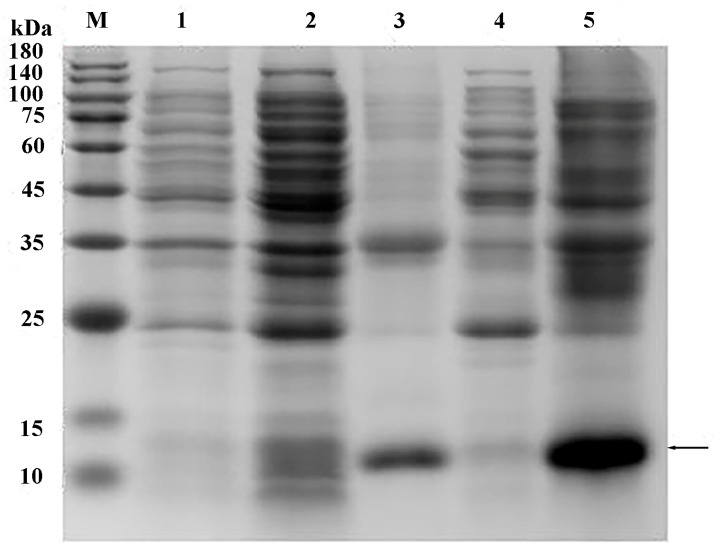
Analysis of SDS-PAGE for the small-scale expression of a fusion protein. M: Protein Marker; 1: Total protein before induction; 2: Supernatant at 20 °C; 3: Precipitate at 20 °C; 4: Supernatant at 37 °C; 5: Precipitate at 37 °C. The black arrow points to the target protein.

**Figure 10:**
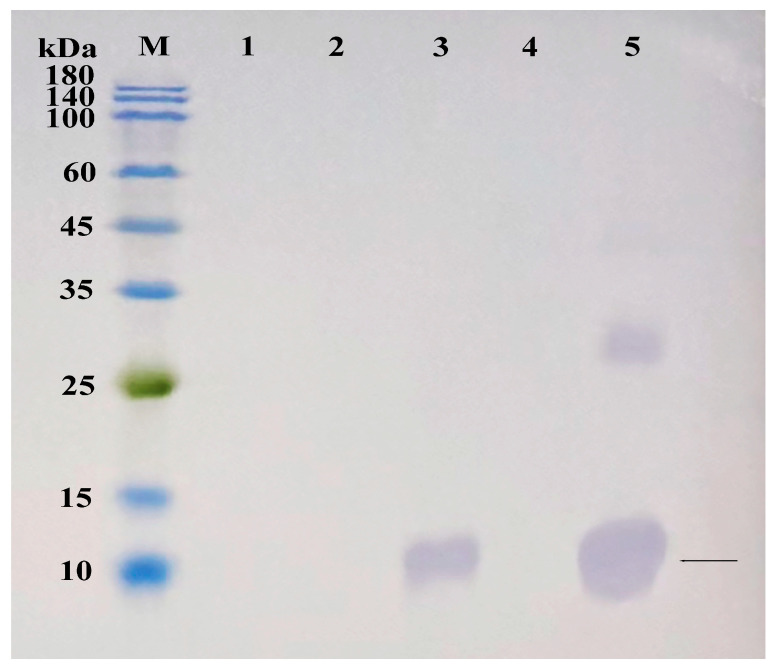
Expression trial of fusion protein Western blot analysis chart. M: Protein marker; 1: Total protein before induction; 2: Supernatant at 20 °C; 3: Precipitate at 20 °C; 4: Supernatant at 37 °C; 5: Precipitate at 37 °C. The black arrow points to the target protein.

**Figure 11:**
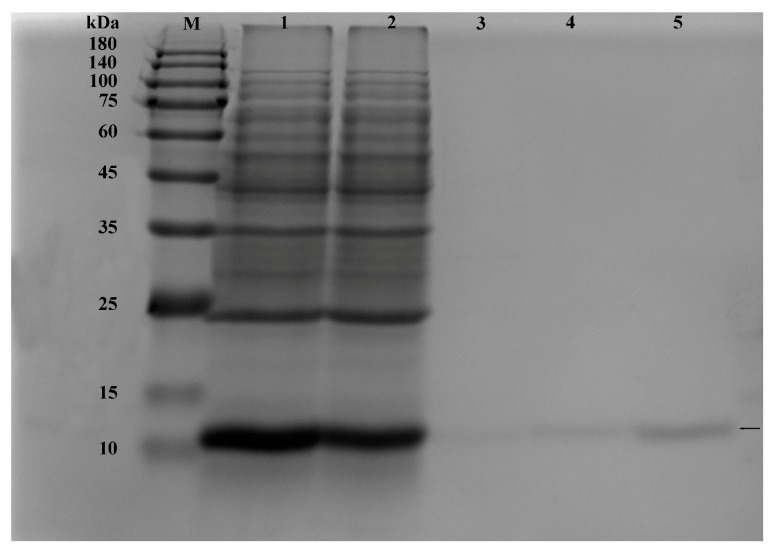
Purification of fusion protein by nickel-affinity agarose chromatography and SDS-PAGE analysis diagram. M: Protein marker; 1: Loading; 2: Flow out; 3: 20 mM Imidazole elution component; 4: 50 mM Imidazole elution component; 5: 500 mM Imidazole elution component. The black arrow points to the target protein.

The *AoOBP8* protein sequence was constructed into the expression vector and then transformed into competent cells of *E. coli*. The cells were cultured and induced to express, and the bacterial cells were collected. The samples were purified for small-scale experiments. The expression and purification effects of the target protein were verified through SDS-PAGE and Western blot. To further determine whether the target protein was expressed, the TMB color development kit was used for color reaction. Following the Western blot procedure, the labeled antibodies were employed for detection (Figure 9, Figure 10 and Figure 11). In conclusion, the target protein is expressed with inclusion bodies, and affinity purification can successfully purify the target protein.

## 4. Discussion

*A. orientalis*, as an efficient and environmentally friendly pest control strategy for *L. delicatula*, has high potential as a biocontrol agent for the suppression of this pest, and it has already been confirmed in the United States, South Korea, and China [9,37,38,39]. Previous studies have demonstrated that olfaction is the most crucial chemical-sensing pathway for parasitic wasps. These insects rely on their highly sensitive olfactory detection system to regulate various behavioral responses, including mating, host seeking, host location, and host selection [40]. Currently, research on the chemosensory genes of various insect species is advancing rapidly; however, the chemosensory genes in parasitic wasps have received comparatively less attention. In this study, we conducted a transcriptomic analysis of *A. orientalis* using the Illumina NovaSeq 6000 high-throughput sequencing platform (San Diego, CA, USA). We successfully obtained 71,513 transcripts and 40,182 unigenes. This marks the first time we have constructed a transcriptome database from the adults of *A. orientalis*, from which we identified a total of 62 chemosensory protein genes (24 OBPs, 4 CSPs, 26 ORs, 3 IRs, 3 GRs, and 2 SNMPs). The assembled sequence data provides a wealth of information for the functional characterization and identification of the *A. orientalis* transcriptome, as well as for further studies on several critical genes. Previous studies have identified chemosensory membrane genes in several parasitic wasps, namely, *Trichogramma chilonis* (85 genes) [12], *C. cunea* (144 genes) [14], *A. confusum* (84 genes) [15], *A. japonicus* (201 genes) [22], *T. remus* (62 genes) [24], *C. vestalis* (253 genes) [41], *Q. mendeli* (124 genes) [42], *Anagrus nilaparvatae* (163 genes) [43], *Aenasius bambawalei* (324 genes) [44], *Aphidius gifuensis* (100 genes) [45], and *Macrocentrus cingulum* (112 genes) [46], based on transcriptome sequencing. In comparison with the genes identified in other parasitic wasps, *A. orientalis* has a relatively small number of chemosensory-related genes; nonetheless, this quantity remains within a reasonable range. Due to the small size of *A. orientalis*, it is challenging to dissect specific tissues such as the antennae and mouthparts. Consequently, this study conducted transcriptome sequencing on whole individuals, including both female and male adults. As a result, some chemosensory genes with low expression levels in the antennae and mouthparts could not be identified. Additionally, transcripts that were present in low abundance and those that were too divergent to be identified through a BLAST search may have been missed during the transcriptome analysis [14]. Therefore, it is unlikely that the identified genes encompass the complete set of chemosensory genes in *A. orientalis*. Nevertheless, this study marks the first comprehensive characterization of chemosensory genes in *A. orientalis*. Our findings offer valuable insights into the molecular mechanisms of chemoreception in this species and highlight potential molecular targets for use in biological control strategies. In this study, the wasps we collected emerged from the host eggs as adults within 24 h, so they had finished mating. We assume that these chemosensory genes with female antennal-specific or dominant expression profiles may play important roles in locating suitable hosts and oviposition sites. This, however, should be verified in further experiments.

OBP and OR have important roles to play in the process of olfaction in insects. Odorant molecules entering through pores interact with OBP/PBPs, which then become solubilized, allowing for the transport of odorants to ORs located in the membrane of the ciliated dendrites [12,47]. A total of 24 OBPs were identified in the adult transcriptomes of *A. orientalis*. The number of OBPs of *A. orientalis* identified was smaller than that of *C. cunea* (25 OBPs) [14], *Q. mendeli* (58 OBPs) [42], and *A. bambawalei* (54 OBPs) [44]. However, it was greater than the number found in *T. chilonis* (22 OBPs) [12], *C. vestalis* (22 OBPs) [16,41], *A. confusum* (11 OBPs) [15], *Sclerodermus* sp. (10 OBPs) [23] and *T. remus* (5 OBPs) [24]. More OBPs were not found because, compared with the antennae, RNA obtained from the whole body may result in genes with low expression that cannot be detected [48]. Furthermore, several genes, including *AoOBP1*, *AoOBP4*, *AoOBP7*, *AoOBP11*, *AoOBP24*, *AoCSP4*, *AoOR12*, *AoOR13*, and *AoSNMP1*, exhibited higher expression levels in male adults than in female adults. Additionally, there are two genes, *AoOR12* and *AoOR13*, that are exclusively expressed in male adults. Based on the higher expression levels of those chemosensory-related genes in males than in females, we suggest that these male-enriched OBPs may be involved in sex-specific behaviors, such as by selectively combining with and transporting some pheromones released by females in the process of molecular recognition, and in searching for suitable mates [49,50]. In contrast, several olfactory genes, including *AoOBP5*, *AoOBP14*, *AoOBP17*, *AoOBP18*, and *AoSNMP2*, showed higher expression levels in female adults compared with males. We hypothesize that these genes, which are highly expressed in female insects, may be involved in their search for suitable hosts and egg-laying sites. This hypothesis is further supported by findings related to *T. remus* [24].

Olfactory receptors (ORs) are crucial for insects to detect general odors and sex pheromones. Most ORs in insects are expressed in the antennae [12,51,52]. In our study, a total of 26 ORs were identified in the transcriptome of *A. orientalis*. The number of identified ORs was lower than the number found in *C. cunea* (80 ORs) [14], *T. chilonis* (45 ORs) [12], *A. gifuensis* (62 ORs) [45], and *C. vestalis* (158 ORs) [41]. There may be several explanations for these differences. As OR expressions are modulated by different environmental conditions in which various types of scents exist, lab-reared *A. orientalis* have no opportunity for exposure to such a diversity of odors or volatiles released from host insects or related plants. For this reason, we speculate that some of the olfactory receptor genes may not be expressed well. Previous studies have shown that some OR genes of *M. mediator* were upregulated following contact with host odors [20]. Additionally, parasitoids may also have different expressions of the OR genes under different physiological situations. Some ORs might be specifically expressed at different developmental stages or in other olfactory tissues [46,53]. Overall, the factors mentioned above may affect the number of ORs identified in *A. orientalis*.

CSPs are soluble carrier proteins that may have a function similar to OBPs in insects [54]. In our study, we only identified four CSPs, which were fewer than the number found in *C. cunea* (11 CSPs) [14], *Sclerodermus* sp. (10 CSPs) [23], *C. vestalis* (11 CSPs) [41], and *A. nilaparvatae* (11 CSPs) [43]. CSPs can be detected in both chemosensory tissues (including the antennae, maxillary palps, labial palps, etc.) and non-chemosensory tissues (including the legs, wings, and pheromone glands) [55]. Our transcriptome analysis showed no significant differences for some CSPs between males and females. This indicates that the CSPs may be involved in odorant detection [55]. However, further experimental verification is needed to determine how this protein functions.

IRs are expressed in sensory neurons with a combinatorial fashion that responds to many distinct odors. In different olfactory neurons, IRs are sufficient to contribute to the responsiveness of ectopic odors [56]. A total of three IRs were identified from the transcriptome in *A. orientalis*. This is less than the amount that was identified in *T. chilonis* (14 IRs) [12], *A. confusum* (19 IRs) [15], and *A. gifuensis* (23 IRs) [45]. The DEG result obtained in our study showed that the expression levels of IR in both male and female adults’ libraries are relatively low. This gives us reason to suggest that the IRs identified in both male and female *A. orientalis* could be involved in the processes of searching for food or avoiding harmful substances. For example, certain odor-evoked behaviors have been related to IR pathways in *Drosophila*; the activity of IR64a, which is acid-sensing, has proven to be both necessary and sufficient to promote the behavioral aversion that allows flies to avoid unripe or rotting fruit [57].

Among insect SNMPs (SNMP1 and SNMP2), SNMP1 is usually expressed in the neuron cells that respond to pheromones, whereas SNMP2 is normally expressed in the supporting cells [58]. In this study, two SNMPs were identified in *A. orientalis*. According to the analysis of the transcriptome, SNMP1 was down-regulated in females in comparison with males, while the opposite was true for SNMP2. This suggests that the function of SNMP1 may also be involved in odor or pheromone processing. This is supported by a study showing SNMP1 to be abundantly expressed and localized to the receptor membrane of sex-pheromone-specific olfactory sensory neurons (OSNs) [59].

Gustatory receptors can recognize non-volatile compounds, including sugars, bitter compounds, and the gas carbon dioxide [60]. This study identified a total of three GRs in *A. orientalis*. This is less than the amount that was identified in *C. cunea* (17 GRs) [14], *Q. mendeli* (10 GRs) [42], and *M. cingulum* (20 GRs) [46]. The expression levels of IRs in both male and female adults’ libraries are relatively low. We speculate that a possible reason is that GR expression levels are quite low and mainly expressed in gustatory organs [61].

OBPs act in insect olfactory processes, and OBPs are expressed in the olfactory organs and play a role in the binding and transport of hydrophobic odorants through the sensillum lymph to olfactory receptor neurons within the antennal sensilla [62]. The identification of chemosensory genes from the transcriptome database is a vital step in studying the chemosensory perception process of parasitic wasps [41]. In this study, the *AoOBP8* gene was cloned from the transcriptome database of *A. orientalis* by reverse transcription-PCR and RACE-PCR, and recombinant *AoOBP8* was expressed in a prokaryotic expression system. In addition, the relative expression level of *AoOBP8* in the head was significantly higher than in the thorax and abdomen. Our results indicate that the head tissues contain many chemical-sensing genes, which play a crucial role in the behaviors of parasitic wasps, such as searching for hosts and identifying hosts. This hypothesis is further supported by findings related to *T. remus*. Most of the olfactory genes in *T. remus* are highly expressed in the heads of both female and male adults [24]. Furthermore, previous studies have shown that odorant-binding protein 8 of *Orius sauteri (OsauOBP8*) could bind with four common insecticides (phoxim, fenitrothion, chlorpyrifos, deltamethrin), and these results help to better understand the molecular mechanisms of OBPs to insecticides in insects [63]. However, whether *AoOBP8* also has similar functions to *OsauOBP8* remains to be verified through further experiments.

Various inorganic compounds play a crucial regulatory role in the processes by which parasitic wasps search for hosts, identify them, mate, and lay eggs. Consequently, the genes responsible for chemical sensing in these wasps are closely linked to their function in biological control. *A. orientalis*, an important parasitic enemy of the forestry pest *L. delicatula* during its egg stage, has been identified as a candidate gene through transcriptome screening. This research offers valuable genetic resources for investigating the interactions between pests, plants, and parasitic wasps at the molecular level. The findings indicate that the genes associated with chemical perception in *A. orientalis* are expressed differently in female and male insects. This suggests that these genes have distinct roles in host recognition, localization, mating, oviposition, and foraging for food. They can serve as target genes for the development of attractants, providing a reference for future in-depth studies on the molecular mechanisms of chemical ecology in *A. orientalis*. Ultimately, this research aims to enhance the utilization of *A. orientalis* for controlling *L. delicatula*.

## 5. Conclusions

*A. orientalis* demonstrates considerable potential for the biological control of *L. delicatula*, which depends on a sensitive olfactory system to identify and locate appropriate hosts for reproduction. The transcriptome database of *A. orientalis* females and males was constructed, and a total of 71,513 transcripts and 40,182 unigenes were identified. Overall, 62 chemosensory genes were screened from the NR database. Phylogenetic analysis showed that these chemosensory genes were most closely related to chemosensory genes found in other Hymenopteran insects. RT-qPCR detection showed that some chemosensory genes showed significant differences and displayed sex-biased expression, indicating that female adults play a key role in the recognition of chemical information for searching for a host and oviposition. In addition, *AoOBP8* was highly expressed in the adult head, indicating that the head plays a key role in the recognition of chemical information. However, further experimental verification is needed to determine how these chemosensory proteins function. The findings herein offer a basis for revealing the chemical–ecological regulatory mechanisms of host finding and oviposition behavior, and provide a reference for developing bio-control methods in forestry.

## Figures and Tables

**Table 1:** Primers used in this study.

Primer Names	Primer Sequences	Length of Primer	Primer Usage
*AoOBP8*-F	GATATTCGTCCTCGTTGG	18 bp	amplification of intermediate fragments
*AoOBP8*-R	ATCTTTCGGCGTAGCATT	18 bp	
*AoOBP8*-R1	GCAGCCGTAGCAATCATCTT	20 bp	5′RACE
*AoOBP8*-R2	ATCAATGGCAGTCTCGTGTA	20 bp	
*AoOBP8*-F1	CTATTAGCGAGTGCAAGGC	19 bp	3′RACE
*AoOBP8*-F2	ACAAATGCTACGCCGAAAG	19 bp	
UPM long	CTAATACGACTCACTATAGGGCAAGCAGTGGTATCAACGCAGAGT	45 bp	Universal Primer
UPM short	CTAATACGACTCACTATAGGGC	22 bp	
NUP	AAGCAGTGGTAACAACGCAGAGT	23 bp	
M13F	GTTGTAAAACGACGGCCAG	19 bp	
M13R	CAGGAAACAGCTATGAC	17 bp	
β-*Actin*-F	GTGCGACGTGGACGTGAGAA	20 bp	RT-qPCR
β-*Actin*-R	AGACGGAGCAAGAGCGGTGA	20 bp	
α-*tubulin*-F	TTTCGACGGAGCTTTGAATGTAG	23 bp	
α-*tubulin*-R	TTGGTGATTTCAGCAACGGATAA	23 bp	
Q-*AoOBP8*-F	CGGCATACCGTACTGAAGTT	20 bp	
Q-*AoOBP8*-R	CTTTCGGCGTAGCATTTGTT	20 bp	

**Table 2:** Summary of sequencing data of six cDNA libraries of *A. orientalis*.

Sample	Raw Reads	Clean Reads	Clean Base (G)	Error Rate (%)	Q20 (%)	Q30 (%)	GC Content (%)
AoF1	54,635,230	51,945,542	7.79	0.01	99.02	97.03	38.015
AoF2	45,726,002	42,760,376	6.41	0.01	98.78	96.14	37.905
AoF3	54,278,948	51,546,124	7.73	0.01	98.94	96.74	37.775
AoM1	67,109,646	64,403,836	9.66	0.01	98.95	96.93	37.175
AoM2	50,562,262	47,874,422	7.18	0.01	98.87	96.62	36.035
AoM3	54,119,120	51,134,978	7.67	0.01	98.89	96.66	36.675

**Table 3:** Illumina transcriptome assembly results for female and male *A. orientalis*.

	Length Range	Transcript	Unigene
Transcript length interval	200~500 bp	27,120	5610
	500~1000 bp	14,422	12,008
	1000~2000 bp	13,241	9938
	≥2000 bp	16,728	12,626
Sequencing Statistics	Total Number	71,513	40,182
	Mean Length	1417	1833
	N50	2692	2904
	N90	563	774
	Total Bases	101,331,167	73,664,248

**Table 4:** Analysis of annotation of a pooled assembly of female and male *A. orientalis* in different databases.

Database	Number of Genes	Percentage (%)
KEGG	22,709	56.52
NR	26,113	64.99
SwissProt	20,542	51.12
TrEMBL	26,326	65.52
KOG	19,036	47.37
GO	23,074	57.42
Pfam	23,512	58.51
Annotated in at least one Database	28,075	69.87
Total Unigenes	40,182	100

## Data Availability

The original contributions presented in this study are included in the article/Supplementary Materials. Further inquiries can be directed to the corresponding author.

## Supplementary Materials

The following supporting information can be downloaded at: https://www.mdpi.com/article/10.3390/insects16111117/s1, Figure S1: Hydropathy plot of *AoOBP8*; Figure S2. Signal peptide of *AoOBP8*; Figure S3. Transmembrane domain of *AoOBP8*; Figure S4. Secondary structure of *AoOBP8*; Figure S5. Tertiary structure of *AoOBP8*; Figure S6. Multiple sequence alignment among *AoOBP8* with OBPs from other parasitoid wasps; Figure S7. Phylogenetic tree of *AoOBP8*; Table S1: DEG analysis of chemosensory-related genes in *A. orientalis*; Supplementary Document S1: Method of RNA Extraction, cDNA Synthesis, Library Construction and Transcriptome Sequencing.
