# Supplementary material for: Transcriptome Characterization and Identification of Chemosensory Genes in the Egg Parasitoid Anastatus orientalis, Along with Molecular Cloning, Sequence Analysis, and Prokaryotic Expression of the Odorant Binding Protein 8 (AoOBP8) from A. orientalis
Source: Insects. 2025 Oct 31;16(11):1117. doi: 10.3390/insects16111117 (PMC12653921; doi:10.3390/insects16111117)
Supplement: Supplementary file 1 [file insects-16-01117-s001.zip › Supplementary Document S1.pdf]

**Supplementary Document S1**

Method of RNA Extraction, cDNA Library Construction and Transcriptome Sequencing

Total RNA from male and female *A. orientalis* was extracted using the Eastep® Super total RNA Extraction Kit (Cat.# LS1040, Shanghai Promega Trading Co., Ltd., Shanghai, China) according to the manufacturer instructions. The concentration and purity of total RNA were measured using a NanoDrop spectrophotometer (Thermo, Wilmington, DE, USA), and RNA integrity was assessed with an Agilent 2100 (Agilent Technologies, Santa Clara, CA, USA) to ensure the samples met the transcriptome sequencing standards. Once the concentration and purity of the total RNA from *A. orientalis* adults were confirmed, library construction was initiated.

The process began by enriching eukaryotic mRNA using Oligo(dT)-coated magnetic beads, then randomly fragmenting the mRNA with fragmentation buffer. The first and second cDNA strands were synthesized using the mRNA as a template, and the cDNA was purified. Following purification, the double-stranded cDNA went through end repair, A-tailing, and adapter ligation. Size selection was performed using AMPure XP beads, and the cDNA library was enriched through PCR amplification. Upon completing the library construction, the initial quantification was performed using a Qubit 3.0 fluorometer (Waltham, MA, USA), with the required concentration ≥ 1 ng/μL. The insert fragments were then examined using the Qsep400 high-throughput analysis system to confirm the expected insert size, to ensure library quality, and Q-PCR was used to accurately measure the effective library concentration (effective concentration > 2 nM).

Once the library passed quality control, sequencing was conducted on an Illumina NovaSeq 6000 high-throughput platform (Illumina, California, USA).

Transcriptome Assembly, Gene Annotation and transcriptome data analysis

Adapter-containing and low-quality reads were removed from the raw sequencing data (raw reads), such as those with more than 10% N bases or those with more than 50% bases and a quality score ⩽ 10. Clean and high-quality data were successfully obtained following a series of quality control procedures. Trinity software (v2.14.0) was applied to split the sequencing reads into shorter fragments (K-mers), which were then extended into longer sequences (contigs). These fragments were assembled into component sets based on overlapping sequences. Using De Bruijn graph methods and sequencing read data, transcript sequences were identified within each component set. A high-quality unigene library was ultimately generated.

Regarding transcriptome data analysis, RPKM (Reads Per Kilobase per Million mapped reads), using the formula RPKM (A) = $(1{,}000{,}000 \times C \times 1{,}000)/(N \times L)$, where RPKM (A) is the expression of chemosensory gene A, C is the number of reads that are uniquely mapped to chemosensory gene A, N is the total number of reads that are uniquely mapped to all unigenes, and L is the number of bases in chemosensory gene A. C represents the number of reads that are mapped to a known set of chemosensory genes determined by the software SOAP(v2.21t) http://soap.genomics.org.cn/. The chemosensory protein genes in A. orientalis were examined for open reading frames (ORFs) using the ORF Finder online tool (https://www.ncbi.nlm.nih.gov/orffinder/ ). The odorant receptor gene sequences of A.

orientalis were subjected to transmembrane domain prediction using the DeepTMHMM online tool (https://dtu.biolib.com/DeepTMHMM )

FPKM（Fragments Per Kilobase of transcript per Million fragments mapped）

$$FPKM = \frac{mapped\ fragments\ of\ transcript}{Total\ Count\ of\ mapped\ fragments\ (Millions) \times Length\ of\ transcript\ (kb)}$$

Method of RNA Isolation and cDNA Synthesis

**Total RNA Extraction Kit (Shanghai Promega Trading Co., Ltd., Shanghai, China) manufacturer's instructions:** Each tube of collected sample was homogenized in an ice bath with 300 μL of lysis solution. Then, 300 μL RNA diluent was added into the tube and mixed well. The sample was heated at 70 °C for 5 min and centrifuged at 4 °C, 14,000× *g*, for 5 min. Then, 500 μL of supernatant was transferred to another new tube, and 250 μL of absolute ethyl alcohol was added and mixed well. The mixture was transferred to a new centrifugal column installed on a collecting pipe and centrifuged at 4 °C, 14000× *g*, for 1 min. At the same time, the filtrate was discarded, and 600 μL RNA lotion was added and centrifuged at 4 °C, 14000× *g*, for 1 min. Fifty μL of the prepared DNase 1 incubation solution was added to the adsorption film center and incubated at room temperature for 15 min. Then, 600 μL RNA lotion was added and centrifuged at 4 °C, 14,000× *g*, for 45 s, repeating the process twice. Meanwhile, the filtrate was discarded. The centrifugal column was installed on the collecting pipe and centrifuged at 4 °C, 14000× *g*, for 2 min. Next, the centrifugal column was anew installed on an elution tube and 100 μL nuclease-free water was added. Solutions were kept at room temperature for 2 min and centrifuged at 4 °C, 14000× *g*, for 1 min. The RNAs were stored at −80 °C. Simultaneously, the concentration and purity of RNA were assayed by NanoPhotometer N60 Touch (IMPLEN GMBH, Munich, Germany) at absorbance ratios of A260/230 and A260/280. The integrity of the total RNA was verified via 1% agarose gel electrophoresis.

**1st Strand cDNA Synthesis Kit (Takara Biomedical Technology (Beijing) Co., Ltd., China) manufacturer's instructions:** In accordance with the manufacturer's instructions, total RNA was reverse-transcribed using the PrimeScript™ II 1st Strand cDNA Synthesis Kit (Takara Biomedical Technology (Beijing) Co., Ltd., China). In other words, 1.0 μL of random 6 mers, 1.0 μL of dNTP mixture and 8 μL of total RNA were mixed to reach 10 μL in the tube, which was then incubated at 65 °C for 5 min to improve reverse transcription efficiency. Then, 4.0 μL of 5×PrimeScript II Buffer, 0.5 μL of RNase Inhibitor and 1.0 μL Primer Script II RTase and RNase-free water was added to reach 20 μL. Finally, the mixture was incubated at 45 °C for 50 min and then incubated at 70 °C for 15 min. The cDNA was stored at −20 °C for subsequent experiments.

The Reaction System for PCR

Each PCR reaction was mixed with 10 μL TB green, 7.8 μL dd$H_2O$, 1.0 μL cDNA, 0.4 μL Rox dye and 0.4 μL of each primer. The thermal cycling profile consisted of an initial denaturation at 95 °C for 5 min and 40 cycles at 95 °C for 10 s and 60 °C for 20 s.
