# Supplementary material for: Transcriptome Characterization and Identification of Chemosensory Genes in the Egg Parasitoid Anastatus orientalis, Along with Molecular Cloning, Sequence Analysis, and Prokaryotic Expression of the Odorant Binding Protein 8 (AoOBP8) from A. orientalis
Source: Insects. 2025 Oct 31;16(11):1117. doi: 10.3390/insects16111117 (PMC12653921; doi:10.3390/insects16111117)
Supplement: Supplementary file 1 [file insects-16-01117-s001.zip › Supplementary Figures(Figure S1-S7).pdf]

Supplementary Figures: Bioinformatics Analyses of *AoOBP8*

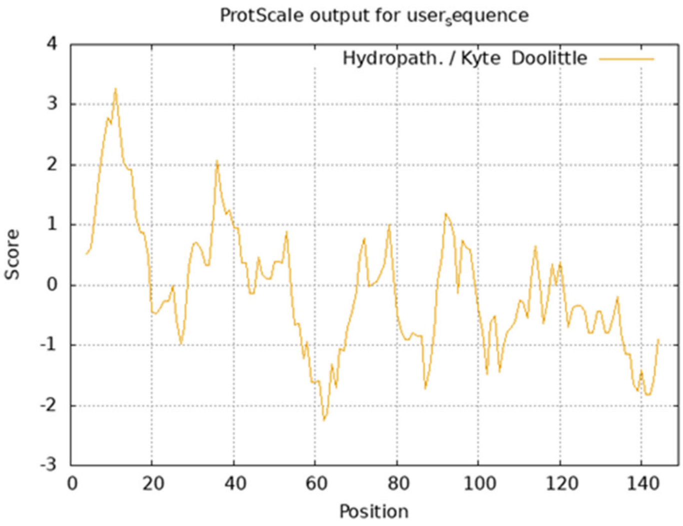

Figure S1. Hydropathy plot of *AoOBP8*.

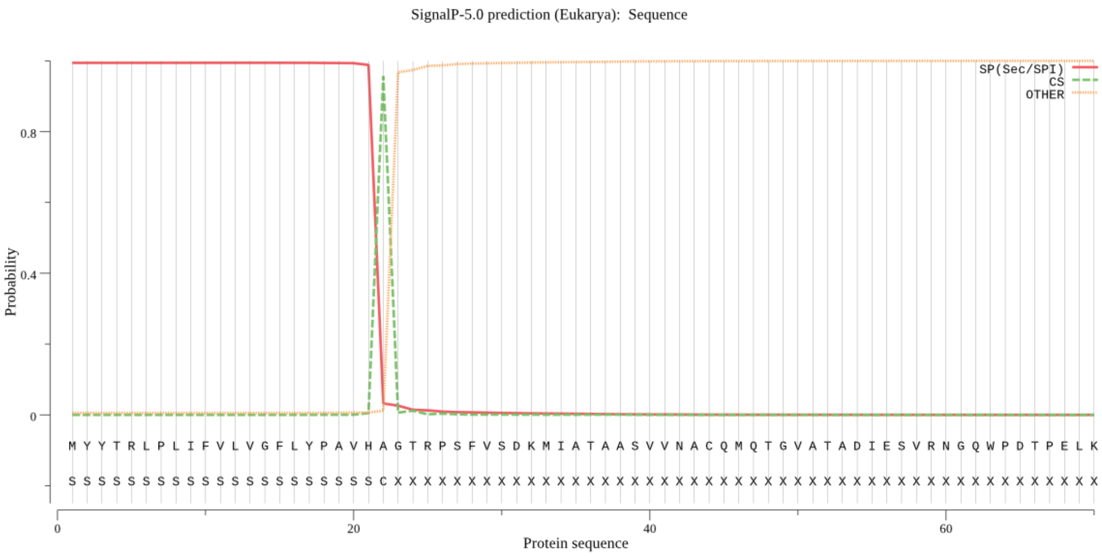

Figure S2. Signal peptide of *AoOBP8* predicted by SignalP-5.0.

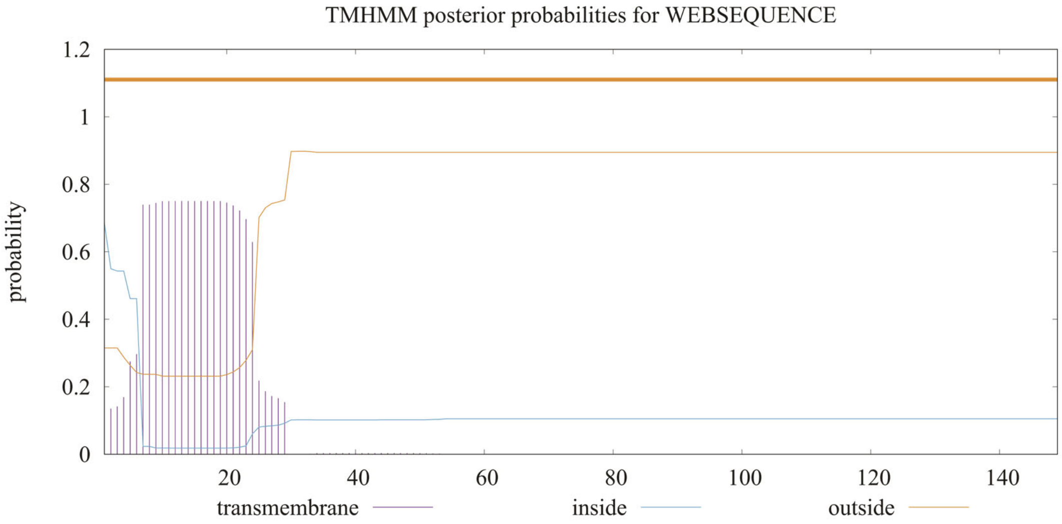

Figure S3. Transmembrane domain of *AoOBP8* analyzed by transmembrane prediction by using the hidden Markov models (TMHMM) program

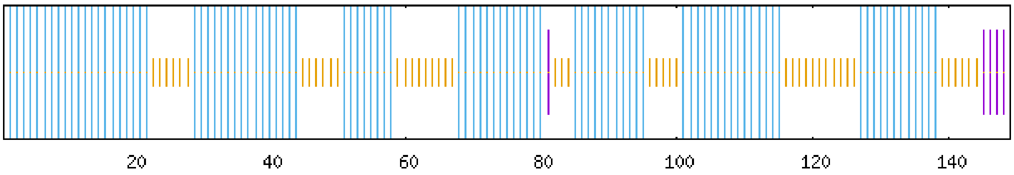

Figure S4. Secondary structure of *AoOBP8* protein in *A. orientalis*. Blue is the helix, purple is the straight chain extension, yellow is the irregular curl.

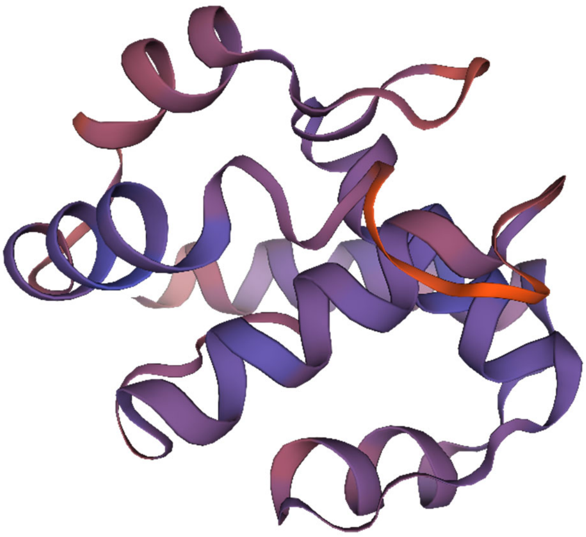

Figure S5. Tertiary structure of *AoOBP8* protein in *A. orientalis*.

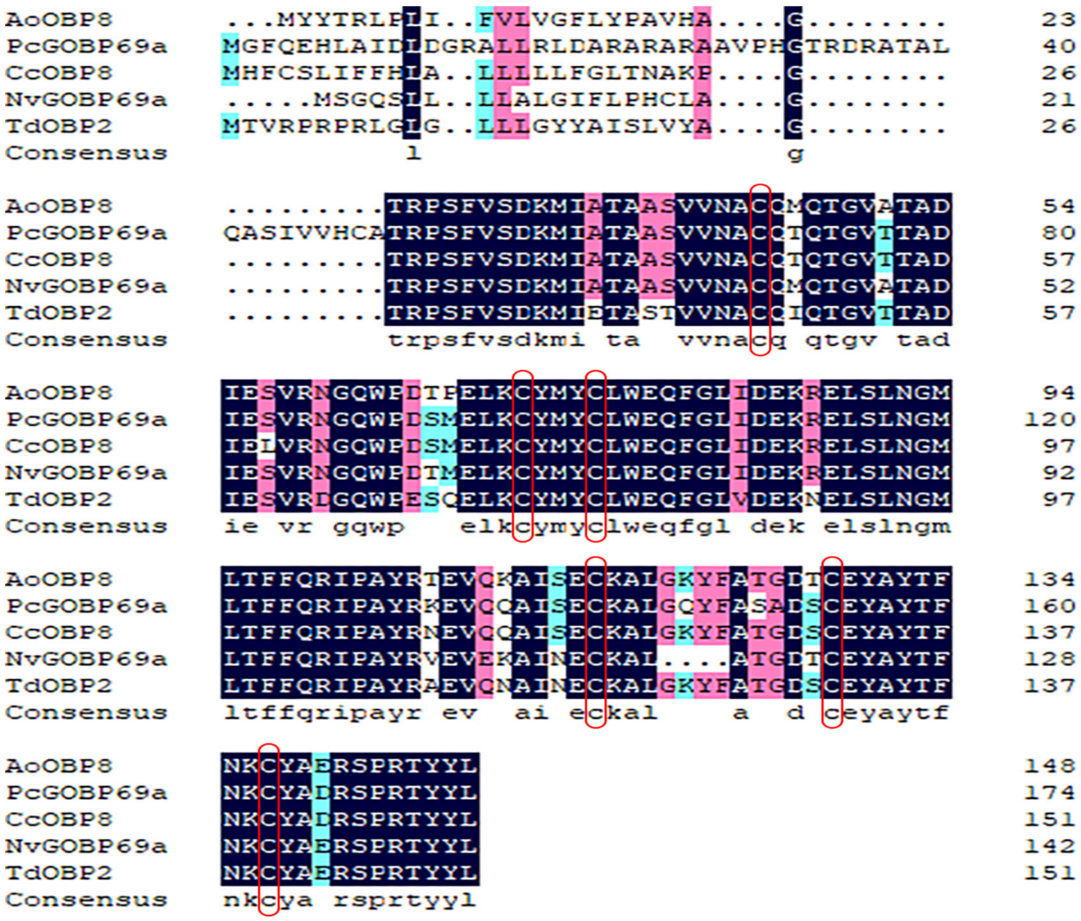

Figure S6. Multiple sequence alignment among *AoOBP8* with OBPs from other parasitoid wasps. Black, pink, blue and white represent 100% identity, identity above 75%, identity above 50% and identity below 30%, respectively. GenBank ID and its corresponding OBP of parasitoid wasps: PQ836618 *A. orientalis* (*AoOBP8*); XP_058792730.1 *Phymastichus coffea* (*PcGOBP69a*); QGW50303.1 *C. cunea* (*CcOBP8*); XP_001606881.1 *N. vitripennis* (*NvGOBP69a*); ANG08492.1 *T. dendrolimi* (*TdOBP2*). The conserved cysteines are indicated in red box.

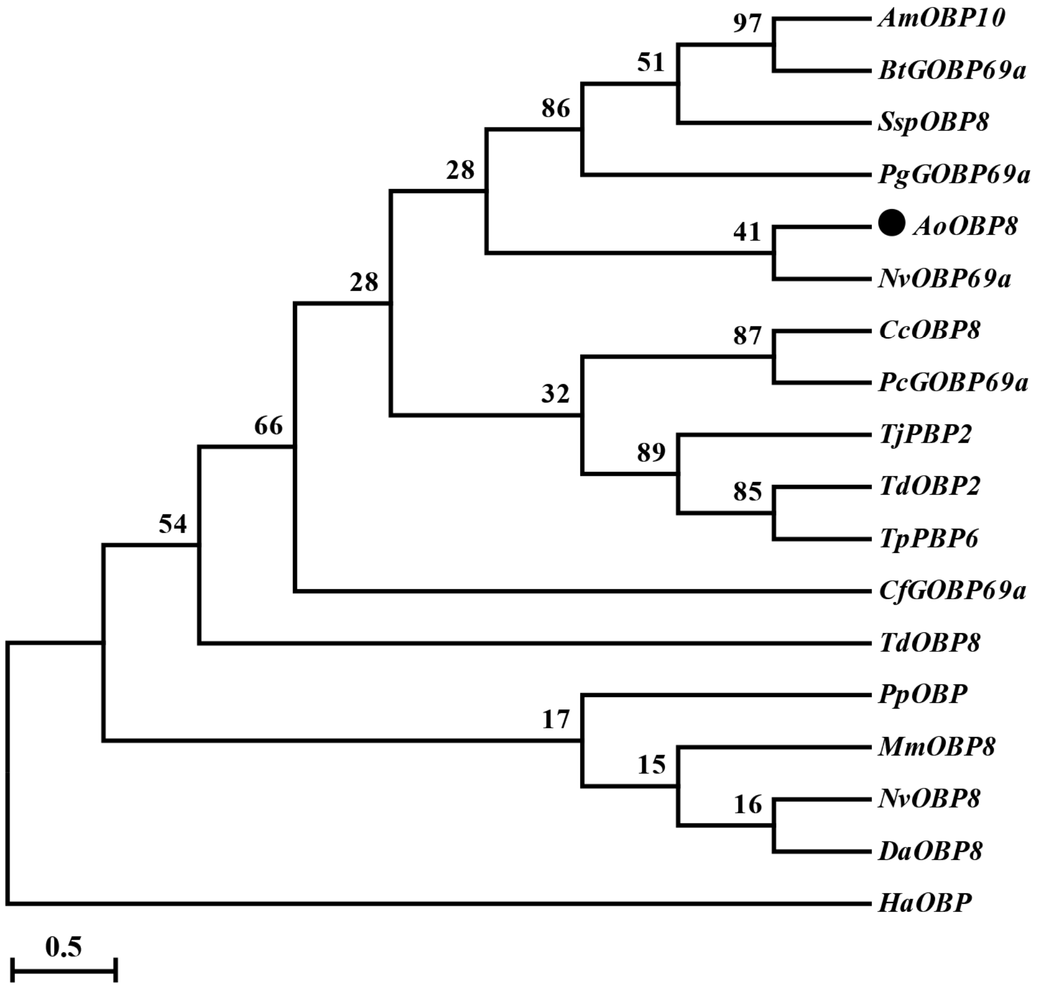

Figure S7. Phylogenetic tree of *AoOBP8* and its homologues from other Hymenoptera insects based on amino acid sequences of OBPs using neighbor joining method. GenBank ID and its corresponding OBPs: PQ836618 *A. orientalis* (*AoOBP8*); XP_006566010.1 *Apis mellifera* (*AmOBP10*); XP_003398556.1 *Bombus terrestris* (*BtGOBP69a*); ALG36141.1 *Sclerodermus* sp. (*SspOBP8*); XP_020298909.1 *Pseudomyrmex gracilis* (*PgGOBP69a*); XP_001606881.1 *N. vitripennis* (*NvOBP69a*); QGW50303.1 *C. cunea* (*CcOBP8*); XP_058792730.1 *P. coffea* (*PcGOBP69a*); ASA40285.1 *Trichogramma japonicum* (*TjPBP2*); ANG08492.1 *T. dendrolimi* (*TdOBP2*); XP_014228437.1 *T. pretiosum* (*TpPBP6*); XP_014206764.1 *C. floridanum* (*CfGOBP69a*); ANG08498.1 *T. dendrolimi* (*TdOBP8*); AJW76495.1 *Pteromalus puparum* (*PpOBP*); AEF14409.1 *Microplitis mediator* (*MmOBP8*); CCD17777.1 *N. vitripennis* (*NvOBP8*); THK33255.1 *Diachasma alloeum* (*DaOBP8*); AEX07278.1 *Helicoverpa armigera* (*HaOBP*).
