# Supplementary material for: Transcriptome Characterization and Identification of Chemosensory Genes in the Egg Parasitoid Anastatus orientalis, Along with Molecular Cloning, Sequence Analysis, and Prokaryotic Expression of the Odorant Binding Protein 8 (AoOBP8) from A. orientalis
Source: Insects. 2025 Oct 31;16(11):1117. doi: 10.3390/insects16111117 (PMC12653921; doi:10.3390/insects16111117)
Supplement: Supplementary file 1 [file insects-16-01117-s001.zip › Supplementary Table (Table S1).pdf]

Table S1. DEG analysis of chemosensory-related genes in *A. orientalis*.

Number	*Gene*	Accession No.	Nucleotide length (bp)	Full length or not	FPKM value		Homology
					male	female	
1	*AoOBP1*	Cluster-19272.0	447	Yes	15.02±4.35	5.24±0.14	odorant-binding protein 17 [*C. cunea*]
2	*AoOBP2*	Cluster-8707.0	1080	Yes	358.31±94.15	395.64±29.46	odorant-binding protein 25 [*Encarsia formosa*]
3	*AoOBP3*	Cluster-13762.0	381	Yes	73.99±18.91	49.03±5.40	odorant-binding protein 28 [*C. cunea*]
4	*AoOBP4*	Cluster-20699.0	417	Yes	262.51±46.21	100.07±5.11	odorant-binding protein 7 [*C. cunea*]
5	*AoOBP5*	Cluster-7499.0	390	Yes	0.13±0.13	8.87±0.72	odorant-binding protein 5 [*E. formosa*]
6	*AoOBP6*	Cluster-28295.1	486	No	42.07±4.87	54.23±2.42	odorant-binding protein 7 precursor, partial [*Procecidochares utilis*]
7	*AoOBP7*	Cluster-20699.1	417	Yes	521.28±66.86	142.92±9.87	odorant-binding protein 7 [*C. cunea*]
8	*AoOBP8*	Cluster-21504.0	450	Yes	18.21±1.88	7.87±0.23	odorant-binding protein 8 [*C. cunea*]
9	*AoOBP9*	Cluster-28295.0	375	No	185.39±12.83	144.80±9.31	odorant-binding protein 7 precursor, partial [*P. utilis*]
10	*AoOBP10*	Cluster-14567.0	390	Yes	6.41±0.28	1.03±0.06	general odorant binding protein 2 [*Quadrastichus mendeli*]
11	*AoOBP11*	Cluster-16217.0	327	Yes	1039.17±91.78	70.21±2.91	general odorant binding protein 4 [*Q. mendeli*]
12	*AoOBP12*	Cluster-21695.1	399	Yes	178.84±43.15	74.96±2.07	general odorant-binding protein 19d-like [*C. floridanum*]
13	*AoOBP13*	Cluster-10987.0	408	Yes	2.01±0.27	9.74±1.82	putative odorant binding protein

							71 [*N. vitripennis*]
14	*AoOBP14*	Cluster-17509.0	390	Yes	1.27±1.27	15.98±1.77	odorant-binding protein 14 [*Trichogramma dendrolimi*]
15	*AoOBP15*	Cluster-14144.0	453	No	43.37±4.95	38.53±7.60	odorant-binding protein 33 [*E. formosa*]
16	*AoOBP16*	Cluster-17374.0	363	Yes	328.85±78.40	207.37±33.74	odorant binding protein 19d-4 [*Bactrocera dorsalis*]
17	*AoOBP17*	Cluster-13101.0	414	No	761.05±9.80	2259.13±100.50	general odorant-binding protein 56d, partial [*P. utilis*]
18	*AoOBP18*	Cluster-19517.0	429	Yes	0.75±0.57	42.61±0.96	PREDICTED: general odorant-binding protein 56d [*Microplitis demolitor*]
19	*AoOBP19*	Cluster-11524.0	405	Yes	591.56±111.00	352.51±18.74	general odorant-binding protein 56d [*N. vitripennis*]
20	*AoOBP20*	Cluster-22311.0	393	Yes	4250.19±376.87	3579.43±250.94	general odorant-binding protein 56d [*C. floridanum*]
21	*AoOBP21*	Cluster-26507.1	366	Yes	1104.09±124.39	929.03±74.66	general odorant-binding protein 56d [*C. floridanum*]
22	*AoOBP22*	Cluster-15652.0	423	No	9.82±2.59	10.08±1.14	general odorant-binding protein 56d, partial [*P. utilis*]
23	*AoOBP23*	Cluster-8438.2	366	Yes	565.77±68.73	33.23±2.96	general odorant-binding protein 72 [*N. vitripennis*]
24	*AoOBP24*	Cluster-23338.0	435	Yes	519.84±38.51	0.08±0.02	general odorant-binding protein 83a [*N. vitripennis*]
25	*AoCSP1*	Cluster-18953.1	357	Yes	71.71±11.11	11.81±1.01	chemosensory protein [*P. utilis*]
26	*AoCSP2*	Cluster-18430.1	387	Yes	0.94±0.29	39.11±1.79	chemosensory protein 2 [*E. formosa*]

27	*AoCSP3*	Cluster-14297.0	387	No	2543.73±294.47	1308.01±49.97	chemosensory protein, partial [*P. utilis*]
28	*AoCSP4*	Cluster-4991.0	387	Yes	209.86±46.49	0.20±0.20	chemosensory protein 4 [*Sclerodermus* sp.]
29	*AoGR1*	Cluster-17956.1	1500	Yes	2.71±0.16	1.28±0.14	gustatory receptor for sugar taste 43a-like [*C. floridanum*]
30	*AoGR2*	Cluster-27863.0	1524	Yes	2.97±0.56	1.11±0.10	gustatory receptor 2 isoform X2 [*N. vitripennis*]
31	*AoGR3*	Cluster-1998.0	2610	Yes	9.10±0.86	3.97±0.16	gustatory receptor 22 [*C. cunea*]
32	*AoIR25a*	Cluster-27530.0	1353	No	0.77±0.12	1.10±0.13	ionotropic receptor 25a isoform X2 [*N. vitripennis*]
33	*AoIR75a*	Cluster-8270.0	1626	No	1.45±0.18	0.80±0.17	ionotropic receptor 75a-like isoform X1 [*N. vitripennis*]
34	*AoIR93a*	Cluster-25127.0	2451	Yes	1.53±0.24	0.65±0.13	ionotropic receptor 93a isoform X3 [*N. vitripennis*]
35	*AoSNMP1*	Cluster-22307.1	1596	Yes	0.42±0.21	16.91±0.94	sensory neuron membrane protein 1 [*N. vitripennis*]
36	*AoSNMP2*	Cluster-27189.0	1566	Yes	15.38±1.90	1.74±0.16	sensory neuron membrane protein 1 [*N. vitripennis*]
37	*AoOR1*	Cluster-20223.0	1422	Yes	13.91±1.10	1.71±0.08	odorant receptor 1 [*N. vitripennis*]
38	*AoOR2*	Cluster-15661.0	1263	Yes	4.16±0.99	0.71±0.10	odorant receptor 2 [*N. vitripennis*]
39	*AoOR3*	Cluster-13415.0	1281	Yes	0.55±0.07	1.14±0.14	odorant receptor 3 [*N. vitripennis*]
40	*AoOR4*	Cluster-15807.0	1260	Yes	1.09±0.15	0.15±0.13	odorant receptor 5 [*N. vitripennis*]
41	*AoOR5*	Cluster-13156.0	1086	Yes	1.71±0.04	0.36±0.07	odorant receptor 17 [*N. vitripennis*]

42	*AoOR6*	Cluster-13156.1	474	Yes	1.99±0.41	0.37±0.09	odorant receptor 17 [*N. vitripennis*]
43	*AoOR7*	Cluster-7358.0	1260	Yes	1.66±0.35	0.04±0.04	odorant receptor 22c-like [*T. pretiosum*]
44	*AoOR8*	Cluster-10869.0	1194	Yes	1.92±0.37	0.17±0.05	odorant receptor 27 [*N. vitripennis*]
45	*AoOR9*	Cluster-24349.0	381	No	4.90±1.21	0.48±0.22	odorant receptor 36, partial [*C. cunea*]
46	*AoOR10*	Cluster-12062.0	1149	Yes	1.80±0.29	0.25±0.04	odorant receptor 60 [*N. vitripennis*]
47	*AoOR11*	Cluster-11545.0	1155	Yes	3.88±0.36	0.36±0.09	odorant receptor 61 [*N. vitripennis*]
48	*AoOR12*	Cluster-2157.1	1209	Yes	4.13±0.55	0	odorant receptor 79 [*N. vitripennis*]
49	*AoOR13*	Cluster-2797.0	1230	Yes	2.55±0.24	0	odorant receptor 79 [*N. vitripennis*]
50	*AoOR14*	Cluster-23617.0	1173	Yes	4.57±0.65	0.25±0.07	odorant receptor 98 [*N. vitripennis*]
51	*AoOR15*	Cluster-20426.0	1125	Yes	0.85±0.29	1.26±0.39	odorant receptor 119 isoform X1 [*N. vitripennis*]
52	*AoOR16*	Cluster-16024.0	1116	Yes	2.49±0.18	2.51±0.46	odorant receptor 122 [*N. vitripennis*]
53	*AoOR17*	Cluster-26162.1	798	Yes	5.70±0.97	1.16±0.08	odorant receptor 142 [*N. vitripennis*]
54	*AoOR18*	Cluster-25648.0	1212	Yes	0.88±0.14	0.19±0.06	odorant receptor 151 [*N. vitripennis*]
55	*AoOR19*	Cluster-9558.0	573	Yes	1.95±0.38	0.42±0.02	odorant receptor 161 [*N. vitripennis*]
56	*AoOR20*	Cluster-9352.0	1173	Yes	2.08±0.06	0.15±0.05	odorant receptor 166 isoform X1 [*N. vitripennis*]
57	*AoOR21*	Cluster-24976.0	1209	Yes	0.24±0.13	1.3±0.16	odorant receptor 191 [*N. vitripennis*]
58	*AoOR22*	Cluster-6135.0	1188	Yes	3.24±0.63	0.03±0.02	odorant receptor 193 [*N. vitripennis*]

59	*AoOR23*	Cluster-11054.0	1164	Yes	1.25±0.16	0.37±0.14	odorant receptor 232 isoform X1 [*N. vitripennis*]
60	*AoOR24*	Cluster-27336.0	1236	Yes	5.79±0.74	0.66±0.26	odorant receptor 236 [*N. vitripennis*]
61	*AoOR25*	Cluster-11488.0	1188	Yes	1.43±0.03	0.15±0.03	odorant receptor 289 [*N. vitripennis*]
62	*AoOR26*	Cluster-28066.0	951	Yes	6.86±1.96	0.41±0.13	odorant receptor 296 [*N. vitripennis*]
